# Enhanced mitochondrial oxidative metabolism in peripheral blood mononuclear cells is associated with fatty liver in obese young adults

**DOI:** 10.1038/s41598-023-32549-w

**Published:** 2023-03-30

**Authors:** Ryosuke Shirakawa, Takayuki Nakajima, Aya Yoshimura, Yukako Kawahara, Chieko Orito, Miwako Yamane, Haruka Handa, Shingo Takada, Takaaki Furihata, Arata Fukushima, Naoki Ishimori, Masao Nakagawa, Isao Yokota, Hisataka Sabe, Satoshi Hashino, Shintaro Kinugawa, Takashi Yokota

**Affiliations:** 1 Department of Cardiovascular Medicine, Faculty of Medicine and Graduate School of Medicine, Hokkaido University, Sapporo, Japan; 2 Health Care Center, Hokkaido University, Sapporo, Japan; 3 Department of Molecular Biology, Faculty of Medicine and Graduate School of Medicine and Institute for Genetic Medicine, Hokkaido University, Sapporo, Japan; 4 Department of Hematology, Faculty of Medicine and Graduate School of Medicine, Hokkaido University, Sapporo, Japan; 5 Department of Biostatistics, Faculty of Medicine and Graduate School of Medicine, Hokkaido University, Sapporo, Japan; 6 Department of Cardiovascular Medicine, Faculty of Medical Sciences, Kyushu University, Fukuoka, Japan; 7 Division of Cardiovascular Medicine, Faculty of Medical Sciences, Research Institute of Angiocardiology, Kyushu University, Fukuoka, Japan; 8 Institute of Health Science Innovation for Medical Care, Hokkaido University Hospital, Kita-14, Nishi-5, Kita-Ku, Sapporo, 060-8648 Japan

**Keywords:** Health care, Risk factors

## Abstract

Systemic inflammation underlies the association between obesity and nonalcoholic fatty liver disease (NAFLD). Here, we investigated functional changes in leukocytes’ mitochondria in obese individuals and their associations with NAFLD. We analyzed 14 obese male Japanese university students whose body mass index was > 30 kg/m^2^ and 15 healthy age- and sex-matched lean university students as controls. We observed that the mitochondrial oxidative phosphorylation (OXPHOS) capacity with complex I + II-linked substrates in peripheral blood mononuclear cells (PBMCs), which was measured using a high-resolution respirometry, was significantly higher in the obese group versus the controls. The PBMCs’ mitochondrial complex IV capacity was also higher in the obese subjects. All of the obese subjects had hepatic steatosis defined by a fatty liver index (FLI) score ≥ 60, and there was a positive correlation between their FLI scores and their PBMCs’ mitochondrial OXPHOS capacity. The increased PBMCs’ mitochondrial OXPHOS capacity was associated with insulin resistance, systemic inflammation, and higher serum levels of interleukin-6 in the entire series of subjects. Our results suggest that the mitochondrial respiratory capacity is increased in the PBMCs at the early stage of obesity, and the enhanced PBMCs’ mitochondrial oxidative metabolism is associated with hepatic steatosis in obese young adults.

## Introduction

Nonalcoholic fatty liver disease (NAFLD) is the most common cause of chronic liver disease. A meta-analysis study revealed that 25% of the adult population worldwide suffers from NAFLD^1^. The prevalence of NAFLD is quite high among obese adults with insulin resistance^2^, and NAFLD also occurs in obese children and adolescents^2^. When NAFLD progresses to nonalcoholic steatohepatitis (NASH) with advanced liver fibrosis, the risk of liver cirrhosis or primary liver cancer becomes much higher, leading to poor clinical outcomes^2^. Moreover, the presence of NAFLD is known to be a major risk for the development of cardiovascular disease^3^. The clinical and economic burdens of NAFLD are increasing with the global pandemic of obesity, and the prevention of NAFLD is thus a national healthcare priority worldwide.

Accumulated evidence suggests that NAFLD is a complex systemic disorder involving insulin resistance, metabolic dysregulation, gut dysbiosis, and inflammation as well as hepatic steatosis^4^. The white blood cell count has been shown to be elevated in individuals with obesity^5,6^, insulin resistance^7^, and NAFLD^8^. Among the white blood cells, the lymphocytes and monocytes (a primary component of peripheral blood mononuclear cells [PBMCs]) secret proinflammatory cytokines, which may cause systemic inflammation in obese subjects^9–11^. However, it has not been known whether an alteration of bioenergetics in PBMCs is related to the progression of NAFLD in obese individuals.

PBMCs highly rely on the mitochondrial oxidative metabolism for the maintenance of their immune activity^12^. Mitochondria play a pivotal role in cellular energy production. Recent advances in the measurement of mitochondrial function have made it possible to less-invasively evaluate the human mitochondrial respiratory capacity by using circulating blood cells^13,14^. We and others have shown that an alteration of mitochondrial function in PBMCs is linked to the pathogenesis or development of a wide variety of diseases such as autism^15^, depression^16^, heart failure^17^, and sepsis^18^.

Here we examined whether the mitochondrial oxidative metabolism in circulating PBMCs is altered in obese young adults, and if so, whether the altered PBMCs’ mitochondrial oxidative metabolism in obesity is associated with systemic inflammation, insulin resistance, and/or NAFLD. We collected PBMCs from obese university students with no comorbidities requiring treatment in order to investigate the possible role(s) of PBMCs’ mitochondrial function in the progression of NAFLD at the early stage of obesity.

## Results

### Baseline characteristics of the obese and control subjects

The baseline data of the obese and control groups are summarized in Table 1. The median ages of the obese and control groups were 24 and 23 years, respectively. As expected, the obese subjects had a greater body weight, body mass index (BMI), waist circumference, and %fat compared to the controls. None of the obese and control subjects had a cardiovascular event history, and none were taking any medication.

**Table 1:** Baseline characteristics of the obese and lean control groups of university students.

	Obese n = 14	Control n = 15
Age, years	24 (22–25)	23 (21–27)
Body weight, kg	99.1 (91.4–105.8)^†^	61.5 (55.6–65.9)
BMI, kg/m^2^	33.9 (31.8–36.0)^†^	21.2 (20.0–23.3)
Waist circumference, cm	106.8 (102.9–108.9)^†^	74.5 (71.0–78.5)
%Fat, %	36.0 (31.7–37.4)^†^	16.8 (14.7–25.8)
SBP, mm Hg	127 (122–133)*	117 (110–127)
DBP, mm Hg	74 (69–82)*	71 (62–73)

Values are median (IQR) or n (%).

*BMI* body mass index, *DBP* diastolic blood pressure, *SBP* systolic blood pressure.

*P < 0.05 and ^†^*P* < 0.01 vs. control.

### Increased number of white blood cells, liver dysfunction, insulin resistance, dyslipidemia, and systemic inflammation in the obese subjects

The white blood cell count and biochemical measurements of peripheral venous blood are summarized in Table 2. The obese group had a significantly increased number of white blood cells including neutrophils and lymphocytes compared to the control group. The serum levels of aspartate transaminase (AST), alanine aminotransferase (ALT), and gamma-glutamyl transpeptidase (γ-GTP) were significantly elevated in the obese subjects, who also presented with insulin resistance characterized by increased fasting insulin and homeostasis assessment model of insulin resistance (HOMA-IR). The total cholesterol, low-density lipoprotein (LDL)-cholesterol, and fasting triglycerides were significantly higher in the obese group whereas high-density lipoprotein (HDL)-cholesterol was significantly lower in the obese group, indicating that the obese subjects had dyslipidemia. The obese group had a significantly higher free fatty acids (FFA) compared to the control group. The serum levels of the systemic inflammation marker, high-sensitive C-reactive protein (hs-CRP), and the proinflammatory cytokines, tumor necrosis factor-alpha (TNF-α) and interleukin (IL)-6 were significantly greater in the obese group compared to the control group.

**Table 2:** Laboratory data.

	Obese n = 14	Control n = 15
WBC		
Total leukocytes, /μL	6250 (5975–7300)^†^	5500 (4400–5900)
Neutrophils, /μL	3318 (3166–4196)*	2932 (2473–3445)
Lymphocytes, /μL	2219 (1879–2387)*	1665 (1438–2107)
Monocytes, /μL	375 (327–460)	308 (270–403)
AST, U/L	31 (25–41)^†^	19 (17–25)
ALT, U/L	59 (44–71)^†^	16 (10–29)
γ-GTP, U/L	49 (38–56)^†^	15 (12–22)
Fasting glucose, mg/dL	87 (79–92)	81 (79–86)
Fasting insulin, μIU/mL	13.8 (10.5–24.6)^†^	4.7 (3.5–5.9)
HOMA-IR	3.2 (2.2–4.9)^†^	1.0 (0.7–1.2)
HbA1c, %	5.3 (5.2–5.5)	5.2 (4.9–5.4)
Total cholesterol, mg/dL	201 (184–217)^†^	173 (146–183)
HDL cholesterol, mg/dL	44 (36–48)^†^	59 (55–74)
LDL cholesterol, mg/dL	131 (111–152)^†^	98 (68–122)
Triglycerides, mg/dL	141 (99–169)^†^	50 (42–69)
FFA, μEq/L	542 (500–613)^†^	350 (244–381)
Hs-CRP, mg/L	1.91 (0.86–3.29)^†^	0.24 (0.08–0.43)
TNF-α, pg/mL	0.89 (0.74–1.16)^†^	0.65 (0.56–0.76)
IL-6, pg/mL	2.0 (1.2–2.4)*	1.2 (0.8–1.6)

Values are median (IQR).

*ALT* alanine aminotransferase, *AST* aspartate transaminase, *BMI* body mass index, *FFA* free fatty acids, *HbA1c* hemoglobin A1c, *HDL* high-density lipoprotein, *HOMA-IR* homeostasis model assessment of insulin resistance, *hs-CRP* high-sensitive C-reactive protein, *IL-6* interleukin-6, *LDL* low-density lipoprotein, *TNF-α* tumor necrosis factor-alpha, *WBC* white blood cell, *γ-GTP* gamma-glutamyl transpeptidase.

*P < 0.05 and ^†^*P* < 0.01 vs. control.

### The mitochondrial oxidative metabolism in PBMCs was increased in the obese subjects

**Figure 1:**
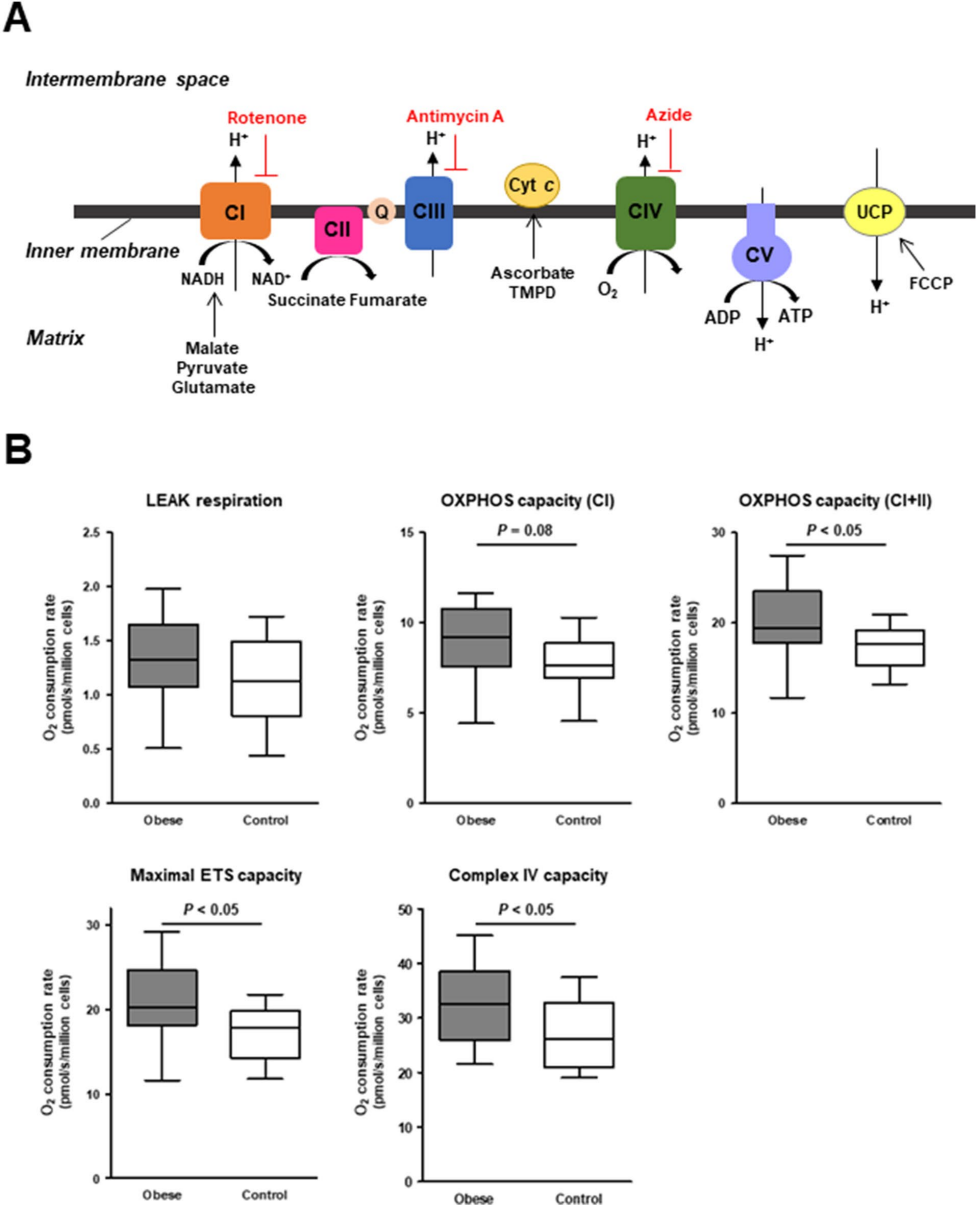
Mitochondrial respiratory capacity in PBMCs. (**A**) Scheme of the mitochondrial electron transfer system (ETS) with the SUIT (substrate–uncoupler–inhibitor–titration) protocol that we used for evaluation of the mitochondrial respiratory capacity in the present study. (**B**) Summarized data of the O_2_ consumption rate during each respiratory state with different respiratory substrates in the permeabilized PBMCs of obese (n = 14) and healthy control subjects (n = 15). The box bounds the interquartile range (IQR) divided by the median, and Tukey-style whiskers extend to a maximum of 1.5 × IQR beyond the box. The LEAK respiration indicates non-ADP stimulated respiration (i.e., state 2 respiration) with CI-linked substrates. The oxidative phosphorylation (OXPHOS) capacity, an ADP-stimulated respiration (i.e., state 3 respiration), was measured in the presence of CI− or CI+II-linked substrates. The maximal ETS capacity was measured after addition of FCCP, an uncoupler, in the presence of CI+CII-linked substrates. The capacity of complex IV was measured after addition of TMPD, an electron donor to cytochrome *c* (cyt *c*), in the presence of ascorbate. *CI* complex I, *CII* complex II, *CIII* complex III, *CIV* complex IV, *CV* complex V, *UCP* uncoupling protein.

We performed a comprehensive measurement of the PBMCs’ mitochondrial respiratory capacity according to the substrate-uncoupler-inhibitor-titration (SUIT) protocol (Fig. 1A). The LEAK respiration did not differ significantly between the obese and control groups, but the oxidative phosphorylation (OXPHOS) capacity with complex I-linked substrates tended to be higher in the obese group and the OXPHOS capacity with complex I + II-linked substrates was significantly higher in the obese group compared to the control group (Fig. 1B). The obese group had a greater maximal electron transfer system (ETS) capacity than the control group (Fig. 1B). The complex IV capacity was also increased in the obese group (Fig. 1B).

### Relationships between NAFLD and increased mitochondrial oxidative metabolism in PBMCs

**Figure 2:**
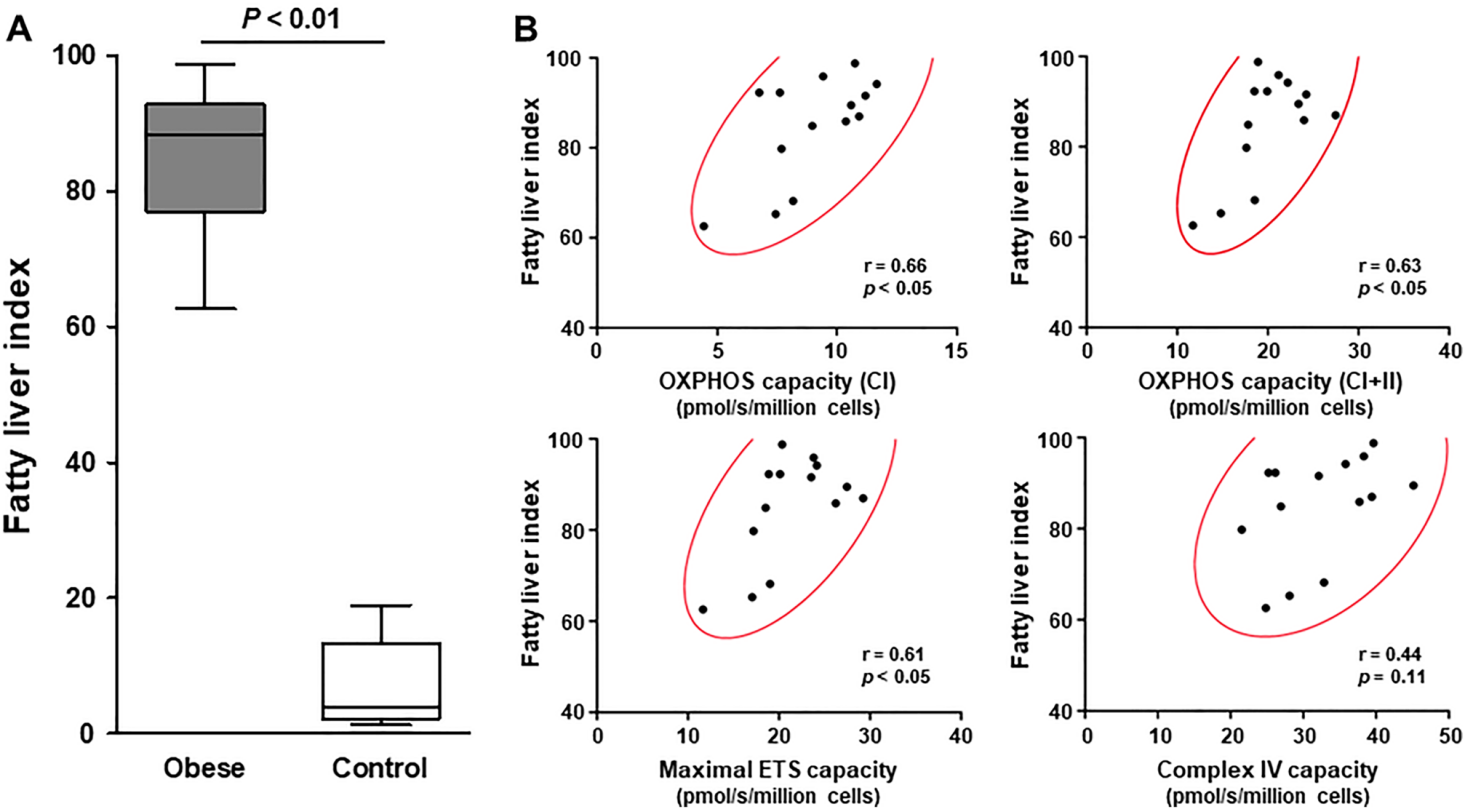
Relationships between fatty liver index and mitochondrial respiratory capacity in PBMCs. (**A**) Summarized data of fatty liver index in obese (n = 14) and healthy lean subjects (n = 15). The *box* bounds the interquartile range (IQR) divided by the median, and Tukey-style *whiskers* extend to a maximum of 1.5 × IQR beyond the box. (**B**) Linear relations between fatty liver index and the mitochondrial respiratory capacity in the PBMCs of obese subjects. A 95% confidence ellipse (*red colored*) is shown in each scatter plot.

All 14 of the obese subjects presented with NAFLD, defined by a fatty liver index (FLI) score ≥ 60 (Fig. 2A). The FLI was positively correlated with the mitochondrial OXPHOS capacity with complex I-linked or complex I + II-linked substrates in PBMCs of the obese subjects (Fig. 2B). In addition, there was a significant positive correlation between the FLI and the maximal ETS capacity in the obese subjects’ PBMCs (Fig. 2B).

### Relationships between insulin resistance or systemic inflammation and increased mitochondrial oxidative metabolism in PBMCs

**Figure 3:**
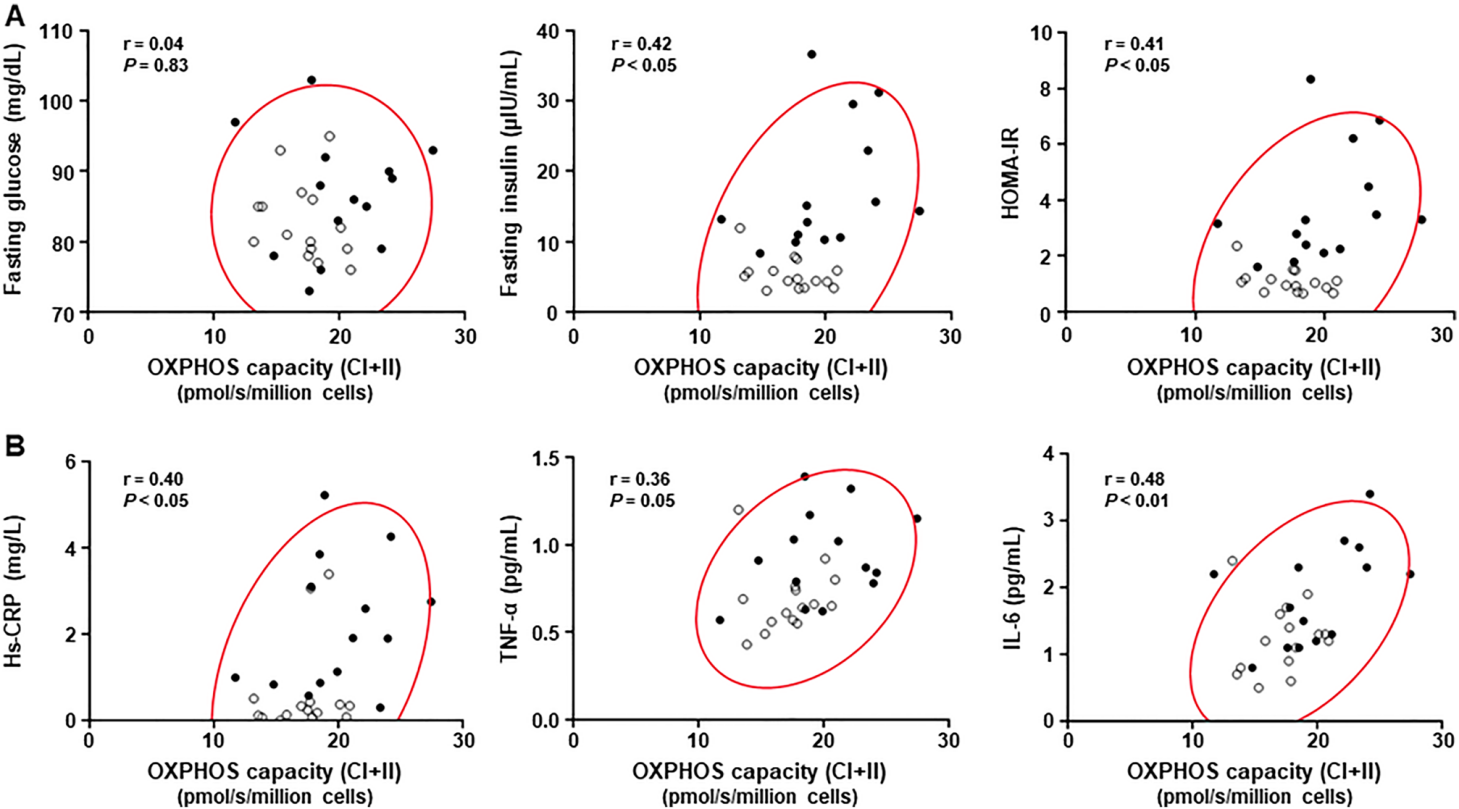
Relationships of mitochondrial respiratory capacity in PBMCs with insulin resistance and systemic inflammation. Linear relations of the mitochondrial OXPHOS capacity with complex I + II-linked substrates (i.e., maximal OXPHOS capacity) in the PBMCs with parameters of insulin sensitivity (**A**) and systemic inflammation (**B**) in all subjects. Obese subjects (n = 14): *black circles*. Healthy subjects (n = 15): *white circles*. A 95% confidence ellipse (*red colored*) is shown in each scatter plot. *OXPHOS* oxidative phosphorylation, *HOMA-IR* homeostasis model assessment of insulin resistance, *hs-CRP* high-sensitive C-reactive protein, *TNF-α* tumor necrosis factor-α, *IL-6* interleukin-6.

In all 29 subjects, the fasting insulin levels and HOMA-IR were positively correlated with the PBMCs' mitochondrial OXPHOS capacity with complex I + II-linked substrates, indicating a relationship between insulin resistance and increased mitochondrial oxidative metabolism in PBMCs (Fig. 3A). In addition, the mitochondrial complex I + II OXPHOS capacity in PBMCs had a modest but significant correlation with serum levels of hs-CRP and IL-6 in the complete series of subjects (Fig. 3B).

## Discussion

This is the first study to document increased mitochondrial OXPHOS capacity with complex I + II-linked substrates in PBMCs from obese young adults compared to lean healthy controls. The PBMCs’ mitochondrial ETS complex IV capacity was also higher in the obese subjects. Notably, the increased PBMCs’ mitochondrial OXPHOS capacity was positively correlated with the FLI score in the obese subjects, indicating that the enhanced mitochondrial oxidative metabolism in PBMCs in obesity is closely linked to hepatic steatosis. Our analyses also revealed a relationship between the increased PBMCs’ mitochondrial complex I + II-linked OXPHOS capacity and (i) insulin resistance and (ii) systemic inflammation.

PBMCs rely on mitochondrial OXPHOS as fuel for the production and release of cytokines and antibodies^19,20^. Since mitochondria are highly sensitive to stress and respond dynamically to changes in the surrounding environment (such as inflammation and oxidative stress), the bioenergetics in circulating PBMCs can be a surrogate marker of metabolic stress^14^. Our present findings demonstrated that the bioenergetics in PBMCs were enhanced in the obese subjects, and this was associated with increased serum levels of hs-CRP and IL-6. Taking the present and previous findings together, we speculate that an activation of the immune system may augment mitochondrial oxidative metabolism in PBMCs to meet a higher energy demand, leading to an excess production of proinflammatory cytokines.

NAFLD is associated with obesity and insulin resistance^21,22^. It is well established that systemic inflammation plays a pivotal role in the development of NAFLD in obese individuals with insulin resistance^23^. In particular, the liver is a key target of circulating proinflammatory cytokines including IL-6, because continuous IL-6 exposure affects hepatic insulin resistance^24^. Our observation of a relationship between the increased PBMCs' mitochondrial OXPHOS capacity and higher FLI values in the obese subjects suggests that enhanced oxidative metabolism in PBMCs may contribute to the progression of NAFLD, possibly via intense systemic inflammation.

Recent advances in the measurement of mitochondrial function have made it possible to investigate alterations of mitochondrial respiratory capacity not only in localized organs and tissues (e.g., liver, heart, and skeletal muscle) but also in cells isolated from small amounts of blood from individuals with various diseases^13,14^. The less-invasive measurement of mitochondrial function using PBMCs (which are an easily available cellular population) is a new research direction with potential applications to explorations of novel biomarkers in some clinical settings^13^. However, no clear data of PBMCs’ mitochondrial function in obese individuals had been available prior to our present investigation. Our obese young-adult subjects with no serious cardiovascular risk factors had an augmented mitochondrial respiratory capacity in PBMCs, which is in contrast to the reports of impaired mitochondrial function in genetic or advanced chronic diseases such as cardiovascular diseases^15–17^, except for the report of increased mitochondrial oxidative metabolism in sepsis^18^.

We speculate that (i) the enhanced mitochondrial oxidative metabolism in PBMCs may reflect a metabolic adaptation to activated immunity at the early stage of obesity, but (ii) continuous metabolic stress and inflammation may eventually cause mitochondrial damage at the advanced stage of obesity, leading to a deterioration of mitochondrial function in PBMCs. Further studies are needed to investigate the pathophysiological role(s) of PBMCs’ mitochondria in obesity-related complications, including NAFLD.

Interestingly, it was reported that obese subjects with NAFLD had upregulated mitochondrial oxidative metabolism in the liver compared to lean healthy subjects^25^, which resembles the mitochondrial respiratory changes that we observed in circulating PBMCs isolated from the obese subjects. The issue of whether a mitochondrial bioenergetic profiling of leukocytes can be an alternative to organ-specific mitochondrial assessments (e.g., liver, heart, and skeletal muscle) remains controversial, as both positive^26^ and negative findings^27^ coexist in this research field. Although the precise underlying mechanisms remain unknown, there may be an interplay between PBMCs and hepatic mitochondria in the progression of NAFLD in obese individuals.

There are some study limitations that should be acknowledged. First, we evaluated NAFLD by the FLI score instead of imaging or histology of the liver; however, the FLI score is recognized as a good surrogate of hepatic steatosis. Second, we did not evaluate mitochondrial function in specific subtypes of PBMCs, including lymphocytes and monocytes. However, an advantage of our study is that the data were obtained from PBMCs, an easily accessed source of peripheral blood cells. Third, we did not evaluate some other confounding factors such as physical activity and dietary calorie intake that may affect the progression of NAFLD. Finally, our study was not designed to elucidate whether an alteration of mitochondrial function in PBMCs is the underlying cause or the consequence of hepatic steatosis in obese individuals.

In conclusion, the results of our analyses demonstrated that the mitochondrial OXPHOS capacity in PBMCs was increased in obese young adults, and it was associated with insulin resistance, systemic inflammation, and hepatic steatosis. Our findings of alterations in circulating blood cells' oxidative metabolism provide important insights into pathophysiology of NAFLD and support the hypothesis that the bioenergetics of PBMCs may be a surrogate marker of metabolic stress in obese individuals.

## Methods

### Study subjects

Fourteen male Japanese subjects with a BMI ≥ 30 kg/m^2^ participated in this study. All of the participants (obese and control groups) were undergraduate or postgraduate university students and were recruited when they presented at the university’s Health Care Center to attend an educational program for lifestyle modification by healthcare providers after the students had undergone an annual physical check-up at Hokkaido University in the years 2016 and 2017. Subjects with chronic inflammatory disease, those treated with an immunosuppressant drug, and heavy drinkers were excluded. As a control group, 15 age- and sex-matched healthy university students with a BMI < 25 kg/m^2^ participated. Written informed consent was obtained from each subject before his participation. This study was approved by the Ethics Committee of Hokkaido University Graduate School of Medicine (No. 16-022) and was registered in the UMIN-CTR (no. UMIN000024737). All investigations conformed to the principles outlined in the Declaration of Helsinki.

### Study protocol

Each of the subjects had a 1-day visit in the morning for a physical examination and an interview regarding his medical history. Peripheral venous blood samples were collected from each subject after a 10 h overnight fast. On the same day within 6 h after blood collection, the mitochondrial respiratory capacity in PBMCs was measured. The rest of blood was stored at − 80 °C for the later analyses of cytokines.

### Body composition measurement

Each subject’s body composition including percent fat was evaluated by a bioimpedance analysis device (InBody 230; Biospace, Seoul, South Korea) as described^6^.

### Laboratory measurements

After the blood collection, the blood cell count and biochemical measurements were performed at an external laboratory (SRL Inc., Tokyo) as described^6^. The hs-CRP, the TNF-α, and the IL-6 were measured by a nephelometric assay (Siemens Healthcare Diagnostic Inc., Erlangen, Germany), an enzyme-linked immunosorbent assay (ELISA; R&D Systems Inc., Minneapolis, MN), and a chemiluminescent enzyme immunoassay (CLEIA; Fujirebio Inc., Tokyo), respectively. The HOMA-IR was calculated using the following equation^28^: fasting blood glucose (mg/dL) × insulin (μIU/mL)/405. NAFLD was diagnosed based on our calculation of the subject’s FLI, a well-validated surrogate of hepatic steatosis, which we defined as an FLI score ≥ 60^29^. The FLI score was calculated using the following equation^29^: FLI = (e^0.953 × loge (triglycerides) + 0.139 × BMI + 0.718 × loge (γ-GTP) + 0.053 × waist circumference − 15.745^)/(1 + e ^0.953 × loge (triglycerides) + 0.139 × BMI + 0.718 × loge (γ-GTP) + 0.053 × waist circumference − 15.745^) × 100.

### Isolation of PBMCs

Peripheral blood (18 mL) was collected from the vein in heparin-containing tubes. A Ficoll-Paque gradient medium (GE Healthcare Life Sciences, Piscataway, NJ) was used to isolate PBMCs according to the manufacturer's protocol. Briefly, whole blood was diluted with calcium and magnesium-free phosphate-buffered saline (PBS) (1: 1.5 ratio) and was layered on the top of density gradient media. Then, PBMCs were separated by centrifugation at 400×*g* for 30 min. After twice wash with PBS, isolated PBMCs were resuspended in ice-cold mitochondrial respiration medium (MiR05; in mmol/L: sucrose 110, K-lactobionate 60, EGTA 0.5, 0.1% bovine serum albumin [BSA], MgCl_2_ 3, taurine 20, KH_2_PO_4_ 10, HEPES 20, pH 7.1).

### Mitochondrial respiratory capacity in PBMCs

The mitochondrial respiratory capacity in PBMCs from each subject was measured with a high-resolution respirometer (Oxygraph-2k; Oroboros Instruments, Innsbruck, Austria) immediately after isolation of PBMCs as described^17^.

After the addition of 2 mL of PBMC suspension (2 × 10^6^ cells/mL) to the chamber of the respirometer, digitonin (2 μmol/L) was added to permeabilize the PBMCs. After stabilization, a SUIT protocol was applied in the following order: (i) malate (final concentration, 2 mmol/L), pyruvate (5 mmol/L), and glutamate (10 mmol/L), (ii) adenosine diphosphate (ADP) (5 mmol/L), (iii) succinate (10 mmol/L increments), (iv) carbonylcyanide p-trifluoromethoxyphenylhydrazone (FCCP; 0.5 µmol/L increments), (v) rotenone (0.5 μmol/L) and antimycin A (2.5 mmol/L), (vi) ascorbate (2 mmol/L) and *N*,*N*,*N*ʹ,*N*ʹ-tert-methyl-p-phenyldiamine (TMPD; 0.5 mmol/L), and (vii) sodium azide (10 mmol/L).

LEAK respiration (i.e., non-ADP-stimulated respiration or state 2 respiration) was evaluated after the addition of malate, pyruvate, and glutamate, all of which are complex I (CI)-linked substrates of the ETS. The OXPHOS capacity (i.e., state 3 respiration) with CI-linked substrates was measured after the addition of ADP. Succinate (a complex II-linked substrate) was then added with a titration protocol until maximal respiration was reached, and the subject’s OXPHOS capacity with complex I + II (CI + II)-linked substrates was then measured.

The maximal ETS capacity with CI + II-linked substrates was measured after the titration of FCCP (an uncoupler). The enzymatic capacity of complex IV was evaluated after the addition of TMPD, an electron donor to cytochrome *c*, in the presence of ascorbate to maintain TMPD in a reduced state. Because of the high level of auto-oxidation of TMPD, sodium azide (an inhibitor of cytochrome c oxidase) was added, and we calculated the difference between the oxygen consumption rate with and without sodium azide as the specific capacity of complex IV. Rotenone (an inhibitor of complex I) and antimycin A (an inhibitor of complex III) were added after the FCCP titration for the evaluation of residual oxygen consumption (non-mitochondrial respiration), which was subtracted from each respiration rate value.

The respiratory rate (i.e., the O_2_ consumption rate) values are expressed as pmol/s/million cells of PBMCs. DatLab software (Oroboros Instruments) was used for the data acquisition and data analysis.

### Statistical analysis

Data are expressed as the median (interquartile range [IQR]) or n (%). We used the Mann–Whitney U-test for continuous variables or the χ^2^-test for categorical variables to compare the data between two groups. We conducted a Pearson’s correlation analysis to determine any linear relationship between continuous variables. All of the statistical analyses were performed using GraphPad Prism 7.0a software (GraphPad Software, San Diego, CA) or JMP Pro ver.16 (SAS Institute, Cary, NC). Statistical significance was defined as a probability (*P*)-value < 0.05.

## Data Availability

The datasets used and/or analyzed during the current study are available from the corresponding author on reasonable request.
