# Author Correction: Enhanced mitochondrial oxidative metabolism in peripheral blood mononuclear cells is associated with fatty liver in obese young adults

**DOI:** 10.1038/s41598-024-57171-2

**Published:** 2024-03-21

**Authors:** Ryosuke Shirakawa, Takayuki Nakajima, Aya Yoshimura, Yukako Kawahara, Chieko Orito, Miwako Yamane, Haruka Handa, Shingo Takada, Takaaki Furihata, Arata Fukushima, Naoki Ishimori, Masao Nakagawa, Isao Yokota, Hisataka Sabe, Satoshi Hashino, Shintaro Kinugawa, Takashi Yokota

**Affiliations:** 1 Department of Cardiovascular Medicine, Faculty of Medicine and Graduate School of Medicine, Hokkaido University, Sapporo, Japan; 2 Health Care Center, Hokkaido University, Sapporo, Japan; 3 Department of Molecular Biology, Faculty of Medicine and Graduate School of Medicine and Institute for Genetic Medicine, Hokkaido University, Sapporo, Japan; 4 Department of Hematology, Faculty of Medicine and Graduate School of Medicine, Hokkaido University, Sapporo, Japan; 5 Department of Biostatistics, Faculty of Medicine and Graduate School of Medicine, Hokkaido University, Sapporo, Japan; 6 Department of Cardiovascular Medicine, Faculty of Medical Sciences, Kyushu University, Fukuoka, Japan; 7 Division of Cardiovascular Medicine, Faculty of Medical Sciences, Research Institute of Angiocardiology, Kyushu University, Fukuoka, Japan; 8 Institute of Health Science Innovation for Medical Care, Hokkaido University Hospital, Kita-14, Nishi-5, Kita-Ku, Sapporo, 060-8648 Japan

Correction to: *Scientific Reports* 10.1038/s41598-023-32549-w, published online 30 March 2023

The original version of this Article contained errors in Figure 1 and its accompanying legend, where the complex II and its surroundings were incorrect. The original Figure 1 and accompanying legend appear below.

**Figure 1:**
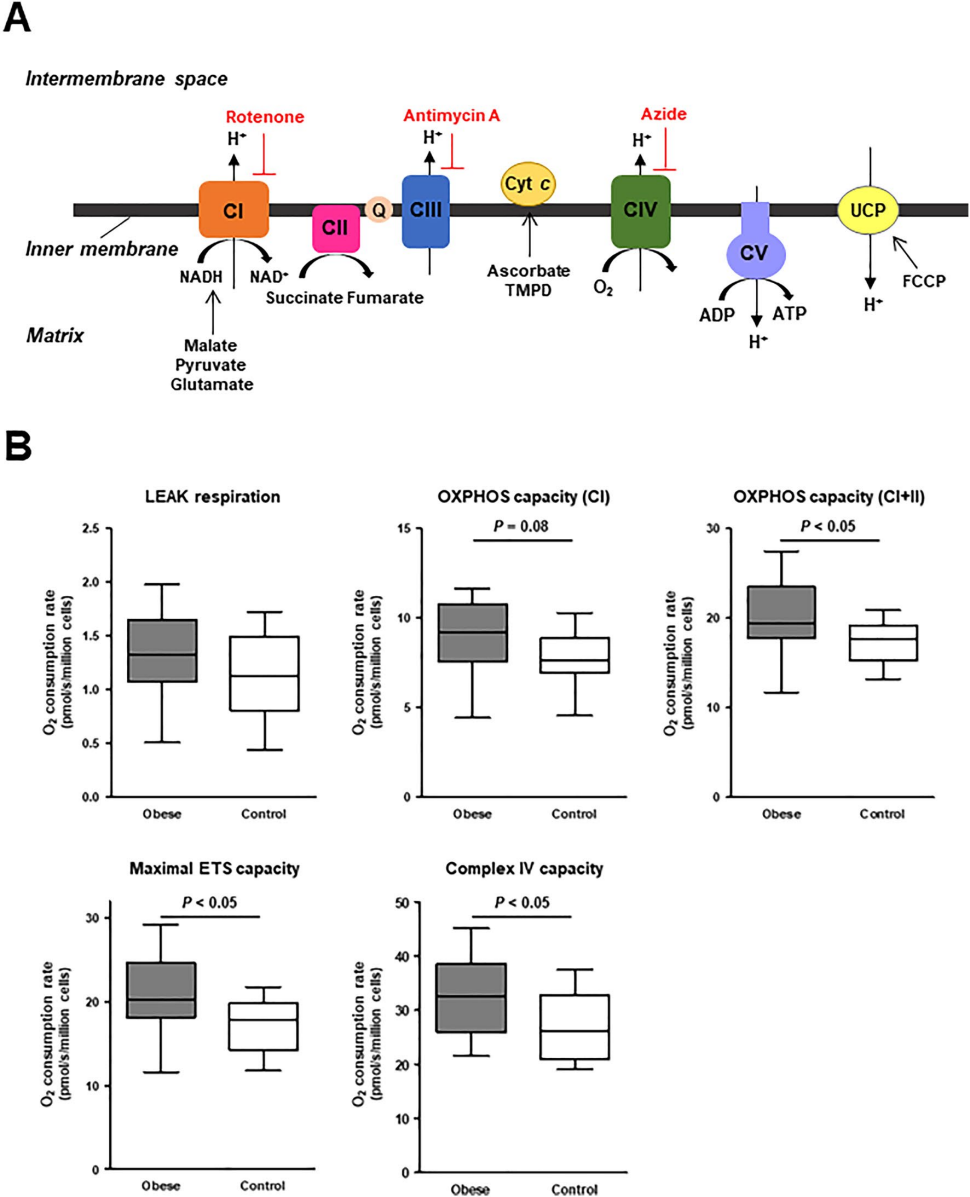
Mitochondrial respiratory capacity in PBMCs. (**A**) Scheme of the mitochondrial electron transfer system (ETS) with the SUIT (substrate–uncoupler–inhibitor–titration) protocol that we used for evaluation of the mitochondrial respiratory capacity in the present study. (**B**) Summarized data of the O_2_ consumption rate during each respiratory state with different respiratory substrates in the permeabilized PBMCs of obese (n = 14) and healthy control subjects (n = 15). The box bounds the interquartile range (IQR) divided by the median, and Tukey-style whiskers extend to a maximum of 1.5 × IQR beyond the box. The LEAK respiration indicates non-ADP stimulated respiration (i.e., state 2 respiration) with CI-linked substrates. The oxidative phosphorylation (OXPHOS) capacity, an ADP-stimulated respiration (i.e., state 3 respiration), was measured in the presence of CI− or CI+ II-linked substrates. The maximal ETS capacity was measured after addition of FCCP, an uncoupler, in the presence of CI + CII-linked substrates. The capacity of complex IV was measured after addition of TMPD, an electron donor to cytochrome *c* (cyt *c*), in the presence of ascorbate. *CI* complex I, *CII* complex II, *CIII* complex III, *CIV* complex IV, *CV* complex V, *UCP* uncoupling protein.

The original Article has been corrected.

